# Elevated SLC1A5 associated with poor prognosis and therapeutic resistance to transarterial chemoembolization in hepatocellular carcinoma

**DOI:** 10.1186/s12967-024-05298-1

**Published:** 2024-06-06

**Authors:** Guixiong Zhang, Yitai Xiao, Jizhou Tan, Hang Liu, Wenzhe Fan, Jiaping Li

**Affiliations:** 1 Department of Interventional Oncology, the First Affiliated Hospital, Sun Yat-Sen University, No. 58 Zhongshan 2 Road, Guangzhou, Guangdong Province 510080 P. R. China; 2 Department of Endoscopy, State Key Laboratory of Oncology in South China, Sun Yat-Sen University Cancer Center, Guangdong Provincial Clinical Research Center for Cancer, Guangzhou, Guangdong Province 510060 P. R. China; 3 Department of Stomatology, the First Affiliated Hospital, Sun Yat-sen University, Guangzhou, Guangdong 510080 China

**Keywords:** Hepatocellular carcinoma, Transarterial Chemoembolization (TACE), SLC1A5, Epithelial-mesenchymal transition (EMT)

## Abstract

**Background:**

Hepatocellular carcinoma (HCC) is a common malignant tumor, and glutamine is vital for tumor cells. The role of glutamine transporter SLC1A5 in tumor progression and transarterial chemoembolization (TACE) efficacy is under study. This research seeks to determine the impact of SLC1A5 expression on the prognosis and TACE efficacy of HCC and elucidate its mechanisms.

**Methods:**

SLC1A5 expression in HCC, correlation with patient outcomes, and response to TACE were studied in an open access liver cancer dataset and confirmed in our cohort. Additionally, the correlation between SLC1A5 expression and hypoxia, angiogenesis and immune infiltration was analyzed and verified by immunohistochemistry, immunofluorescence and transcriptome sequencing. Liver cancer cell lines with SLC1A5 expression knockdown or overexpression were constructed, and cell proliferation, colony formation, apoptosis, migration and drug sensitivity as well as in vivo xenograft tumor were measured. A gene set enrichment analysis was conducted to determine the signaling pathway influenced by SLC1A5, and a western blot analysis was performed to detect protein expression alterations.

**Results:**

SLC1A5 expression was higher in HCC tissue and associated with poor survival and TACE resistance. Hypoxia could stimulate the upregulation of glutamine transport, angiogenesis and SLC1A5 expression. The SLC1A5 expression was positively correlated with hypoxia and angiogenesis-related genes, immune checkpoint pathways, macrophage, Tregs, and other immunosuppressive cells infiltration. Knockdown of SLC1A5 decreased proliferation, colony formation, and migration, but increased apoptosis and increased sensitivity to chemotherapy drugs. Downregulation of SLC1A5 resulted in a decrease in Vimentin and N-cadherin expression, yet an increase in E-cadherin expression. Upregulation of SLC1A5 increased Vimentin and N-cadherin expression, while decreasing E-cadherin. Overexpression of β-catenin in SLC1A5-knockdown HCC cell lines could augment Vimentin and N-cadherin expression, suppress E-cadherin expression, and increase the migration and drug resistance.

**Conclusions:**

Elevated SLC1A5 was linked to TACE resistance and survival shortening in HCC patients. SLC1A5 was positively correlated with hypoxia, angiogenesis, and immunosuppression. SLC1A5 may mediate HCC cell migration and drug resistance via Epithelial-mesenchymal transition (EMT) pathway.

**Supplementary Information:**

The online version contains supplementary material available at 10.1186/s12967-024-05298-1.

## Background

Liver cancer represented the sixth most common cancer type and the third leading cause of cancer-related death in worldwide [1]. Among them, hepatocellular carcinoma (HCC) is the most common, accounting for 75–85% of all liver cancer cases [2]. China accounts for half of the world’s new cases and liver cancer-related deaths, which is the heaviest burden of liver cancer across the world [3, 4]. Although liver resection remains the primary strategy for treatment of most patients with early-stage HCC, there is still a high recurrence rate after undergoing radical liver resection, making their overall prognosis far from satisfactory, with a 5-year survival rate of only 12.1% [5]. The BRIDGE study demonstrated that transcatheter arterial chemoembolization (TACE) is not only the initial course of treatment for HCC patients with unresectable local lesions and adequate liver function, but it can also be used in advanced HCC [6–9]. Post-operative adjuvant TACE and post-recurrence TACE are common therapeutic strategies given the high recurrence rate of HCC following surgical resection [10, 11]. Despite TACE achieving an objective tumor response rate of over 60%, approximately 40% of patients do not respond to the therapy [12, 13]. This is because TACE is likely to induce a hypoxic microenvironment, which leads to angiogenesis and immunosuppression, promoting the recurrence and metastasis of residual cancer cells and impeding the long-term efficacy of TACE [14–17]. Our previously published study had demonstrated that SPP1 + tumor-associated macrophages (TAMs) exhibit a tumor-induced angiogenesis phenotype following TACE therapy, which could be a crucial contributor to tumor recurrence and poor patient survival outcomes [18]. In addition to that, hypoxia could result in various changes in the metabolism of cancer cells, which includes but is not limited to an increase in the utilization of glutamine [19]. The compound glutamine, found in a significant amount within the human body, is the second most prevalent energy source besides glucose and plays a crucial role in numerous metabolic pathways. The demand for glutamine is high in individuals afflicted with cancer, and it is pivotal for tumor cell proliferation and survival. This phenomenon, often referred to as “glutamine addiction,” highlights the vital role glutamine plays in the development and progression of cancer [20, 21]. The SLC1A5 (Solute Carrier Family 1 Member 5) transporter is a protein located on the plasma membrane of cells that is responsible for the transport of glutamine into the cell, and it has been identified as a crucial mediator of the growth and survival of numerous types of cancer cells [22–26]. Unfortunately, the current body of research on the SLC1A5 and its involvement in the development of hepatocellular carcinoma remains quite limited.

This research project aims to investigate the expression of the glutamine transporter, SLC1A5, in hepatocellular carcinoma, and further explore its relationship with the efficacy of TACE. It is also proposed to investigate the potential mechanisms through which SLC1A5 affects the therapeutic outcome of TACE. This may provide an empirical basis for further exploration of the integration of TACE with targeted tumor metabolic therapy.

## Materials and methods

### Data information and processing

The collected clinical and transcriptomic data of HCC patients were from the datasets The Cancer Genome Atlas (TCGA-LIHC, comprising 374 HCC and 50 normal tissue samples), International Cancer Genome Consortium (ICGC-LIRI-JP, comprising 240 HCC and 202 normal tissue samples), and GSE14520 (comprising 222 HCC and 212 normal tissue samples). The inclusion criteria for survival analysis were as follows: (1) samples derived from primary liver cancer pathologically confirmed as HCC; (2) availability of transcriptomic RNA data and complete clinical data (including age, gender, staging, and follow-up time of 30 days or more). All clinical data was matched with the corresponding transcriptomic data. A total of 343 HCC samples from the TCGA-LIHC dataset, 229 from the ICGC-LIRI-JP dataset, and 220 from the GSE14520 dataset were included in the survival analysis. The clinical characteristics of the patients are listed in Supplementary Table S1. The analysis and plotting were performed using the R package “survival” and “survminer”.

The GSE104580 dataset (comprising 81 responders and 66 non-responders HCC patients and related transcriptomic data) was procured to scrutinize the connection between SLC1A5 and TACE effectiveness. Survival analysis was conducted on a total of 146 HCC patients who underwent TACE treatment in the ICGC and GSE14520 datasets. A total of 74 HCC patients (including 19 patients who underwent surgical resection after TACE and 55 patients who underwent TACE because of recurrence after surgical resection) who underwent TACE from January 2015 to July 2021 in the First Affiliated Hospital of Sun Yat-sen University were chosen to verify the association between SLC1A5 and TACE effectiveness. Among them, the transcriptome sequencing data of 12 HCC patients were also acquired to validate the relationship between SLC1A5 and TACE treatment. This study is a retrospective analysis and has been endorsed by the Ethics Committee of the First Affiliated Hospital of Sun Yat-sen University. TACE procedure was conducted by a doctor with more than 10 years of experience and vice senior or above in this department.

### Analyzing correlation of SLC1A5 with hypoxia, angiogenesis, and immune infiltration

The gene set encompassing the hallmark.gmt dataset from the MSigDB database was employed as the functional gene set to assess the pathways associated with TACE resistance using the Gene Set Enrichment Analysis (GSEA) tool GSEA 4.1.0. Transcriptomic data were utilized to investigate the expression correlation of SLC1A5 with hypoxia and angiogenic genes, which was subsequently validated via immunohistochemistry, immunofluorescence (detailed methodologies are provided below) and transcriptome sequence. Moreover, Single sample gene set enrichment analysis (ssGSEA) was executed using the R package “gsva” to explore the association of SLC1A5 expression with the infiltration fraction of 16 immune cells and the activity of 13 immune-related pathways. Furthermore, the relationship among hypoxia, glutamine metabolism, and SLC1A5 was also examined according to the ssGSEA score.

### Mechanism and pathway analysis

The hallmark.gmt and kegg.gmt dataset from the MSigDB database was used as the functional gene set to analyze the pathways related to SLC1A5 by GSEA. And Western blot was used to verify the expression changes of related molecular signaling pathway proteins.

### Immunohistochemistry and Immunofluorescence

The procedure for immunohistochemical staining was similar to the previously described method. Briefly, HCC sample slides were initially deparaffinized and dehydrated by sequential immersion in xylene, ethanol, and distilled water. For antigen retrieval, citrate buffer was used in a microwave at medium power. After blocking with goat serum at room temperature, the tissues were sequentially incubated with a SPP1 antibody (1:1000, 22952-1-AP; Proteintech, Wuhan, China) or SLC1A5 antibody (1:1000, 20350-1-AP; Proteintech) and a secondary antibody. A slide scanner (3DHistech Ltd., Budapest, Hungary) was employed to capture images. The staining intensity, expressed as the H-score (range: 0 ∼ 300), was automatically quantified using Pannoramic Viewer software. For immunofluorescence, the process was identical to immunohistochemistry, except that after incubation with one of the primary and secondary antibodies, CY3 or FITC-labeled TSA was added, respectively. Finally, the nuclei were stained with DAPI. After washing, the sample slides were examined by confocal laser scanning microscopy.

### Cell culture and transfection

All human derived liver cancer cell lines (HepG2, Hep3B, Huh7, HCC-LM3) used in this study were obtained from the laboratory of the First Affiliated Hospital of Sun Yat-sen University. HepG2, Hep3B, Huh7, and HCC-LM3 cells were all grown in Dulbecco’s modified Eagle’s medium (DMEM) with high-level glucose, whereas L02 cells were grown in RPMI-1640 medium. All cell culture media were supplemented with 10% fetal bovine serum and 1% penicillin-streptomycin. All cells were maintained at 37℃ under 5% (v/v) carbon dioxide and used when they reached the logarithmic growth phase. For gene knockdown of SLC1A5, the lentivirus vector containing specific shRNA (GeneChem, Shanghai, China) was employed for stable transduction. Three target sequences were used to increase the reliability of knockdown results and exclude non-specificity (SLC1A5-si1 (CTGAGTTGATACAAGTGAA), SLC1A5-si2 (AGTCCTTGGACTTCGTAAA), and SLC1A5-si3 (TGCTTATCCGCTTCTTCAA)).

### Immuno-blotting (Western blot) and quantitative RT-PCR

Cellular protein was extracted using RIPA lysis buffer (Beyotime, Shanghai, China) and the concentration was determined using a BCA kit (Beyotime). The protein samples were separated via SDS-PAGE gel electrophoresis and transferred onto PVDF membrane. Protein detection was performed using anti-SLC1A5 (Abcam, ab187692), anti-GAPDH (Proteintech, 60004-1-Ig), anti-β-catenin (Proteintech, 51067-2-AP), anti-N-cadherin (Proteintech, 66219-1-Ig), anti-E-cadherin (CST, #14,472), and anti-Vimentin antibody (CST, #5741). ECL chemiluminescence detection reagent was used to visualize the protein bands. Total RNA was isolated using TRIzol-Coloroform method (Vazyme, Jiangsu, China) following the manufacturer’s protocol. RNA concentration was measured using the A260/A280 ratio (Nanodrop 2000 spectrophotometer). Reverse transcription was carried out using PrimeScript RT Reagent Kit (Takara, Shiga, Japan). RT-qPCR was tracked using SYBR Green PCR Reagent (Takara) and performed on a PCR instrument (Bio-Rad, USA). The relative gene expression levels were calculated using the 2-ΔΔCt comparative method with β-actin (ACTB) as an internal control. The primer sequences of SLC1A5 were: Forward primer: 5’-TCATGTGGTACGCCCCTGT-3’; Reverse primer: 5’-GCGGGCAAAGAGTAAACCCA-3’. The primer sequences for β-actin (ACTB) were: Forward primer: 5’-GGCACCAGCACAATGAAG-3’; Reverse prime: 5’-CGATCCACCGGAGTACTTG-3’.

### Proliferation, clone, migration and apoptosis

**Cell proliferation assay**: A total of 2 × 10^3 cells were seeded into 96-well plates and allowed to adhere for 24, 48, 72, and 96 h, respectively. Following this, 100 µL of medium containing 10% CCK8 was added into each well and incubated for a further 2 h. The optical density (OD) was then measured at 450 nm using a Microplate spectrophotometer. **Colony formation assay**: A total of 1 × 10^3 cells were seeded into 6-well plates and allowed to proliferate until visible clones emerged. The cells were then stained with crystal violet solution (Beyotime) and the number of colonies was counted macroscopically. **Wound-Healing Assay**: Transfected cells were seeded into 6-well plates and cultured until reaching greater than 80% confluence. A linear wound was subsequently created by a sterile pipette tip and the cells were incubated in serum-free medium for 24, 48, and 72 h, respectively. The migration of cells during healing was observed under an inverted microscope and quantified by measuring the wound width rate. **Detection of apoptosis by flow cytometry**: Harvested cells were washed with PBS and subsequently treated with 500 µl binding buffer, 5 µl Annexin V-PE, and 5 µl 7-AAD Staining Solution (Vazyme). Following a 15-minute incubation at room temperature in the dark, the samples were examined using a flow cytometry system.

### In *vivo* xenograft tumor assay and drug sensitivity

The animal experiments were approved by the Animal Welfare and Ethics Committee of the First Affiliated Hospital of Sun Yat-sen University. The BALB/c nude mice (female, 4 weeks old, weighing 16 ∼ 18 g) were purchased from Guangzhou Yancheng Biotechnology Co., Ltd. A total of 15 BALB/c nude mice were randomly divided into divided into SLC1A5-NC, SLC1A5-si1 and SLC1A5-si2 group. 5 × 10^6 cells were implanted subcutaneously at the right flanks of nude mice and the mice were randomly divided into different groups (5 mice a group). The calculation formula for tumor volume (TV): TV = length × width^2^/2. The 2 × 10^3 cells were seeded into 96-well plates and cultured for 48 h. Then, the medium was changed into the medium containing different concentrations of fluorouracil (0, 25, 50, 100, 200, 400 µM), oxaliplatin (0, 2.5, 5, 10, 20, 40 µM) or doxorubicin (0, 0.25, 0.5, 1, 2, 4 µM), and continued to be cultured for 1 to 2 days. Finally, CCK8 assay was used to detect cell viability.

### Statistical analysis

Data management and statistical analysis were performed using the R software (version 4.1.0) and GraphPad Prism (version 8.3.0). Gene expression between two groups was compared by either Wilcoxon test or paired t test. Survival curves were plotted using the Kaplan-Meier method and compared by log-rank test. A p value less than 0.05 was considered statistically significant. Unless otherwise specified, all experiments were conducted in triplicate. *p < 0.05, **p < 0.01, ***p < 0.001, and ****p < 0.001.

## Results

### SLC1A5 is highly expressed in HCC and correlates with prognosis

**Fig. 1:**
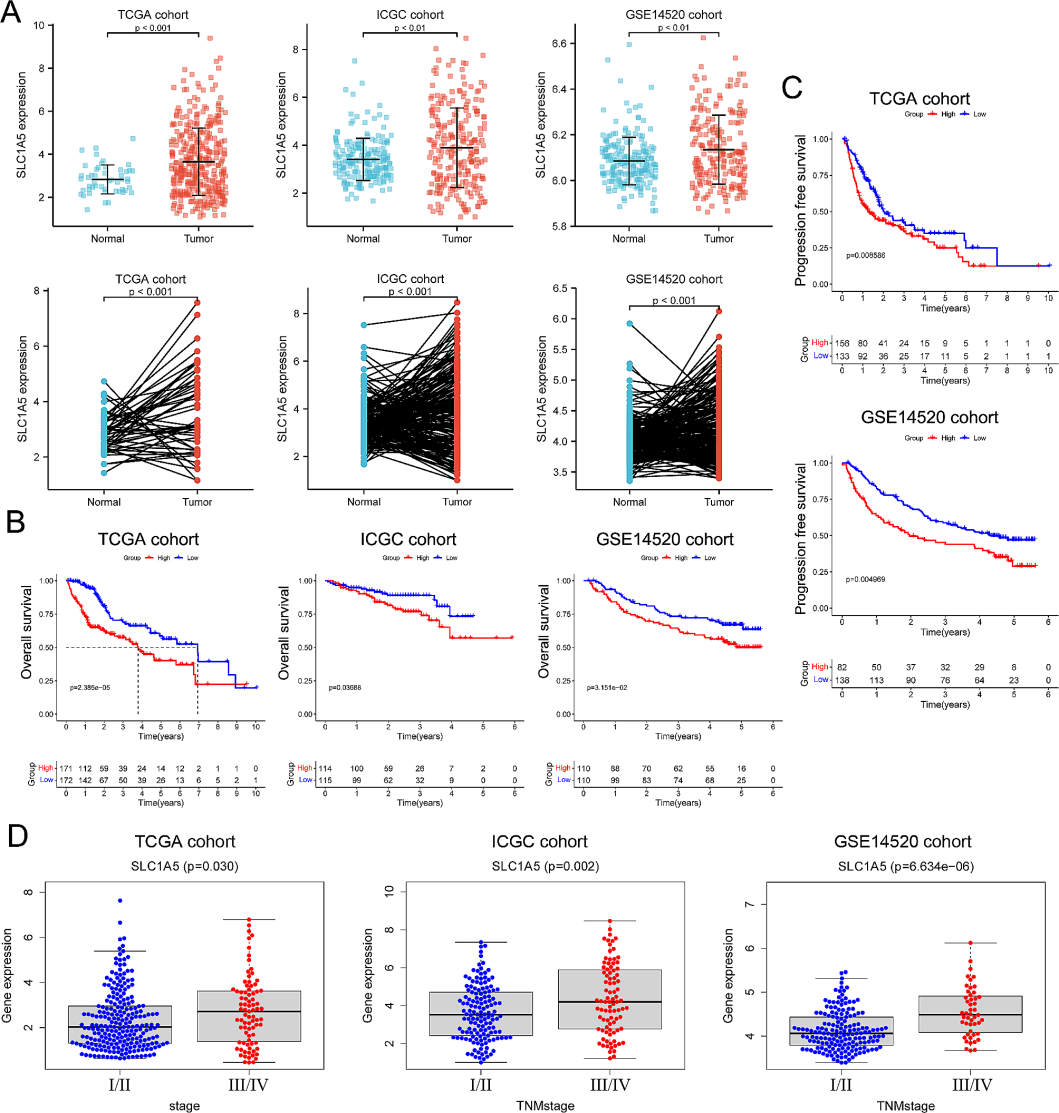
The expression of SLC1A5 in hepatocellular carcinoma and its relationship with survival. (**A**) The overall comparison (upper) and paired comparison (lower) between tumor and adjacent normal tissues. (**B, C**) Kaplan–Meier curves for OS and PFS in different group. (**D**) The expression of SLC1A5 in different TNM stage

The mRNA Differential Expression Analysis indicated that the expression of SLC1A5 in hepatocellular carcinoma (HCC) tissue was substantially higher than in the adjacent normal tissue (*p* < 0.05) (Fig. 1A). The survival analysis indicated that SLC1A5 showed a statistically significant negative correlation with overall survival (OS) and progression-free survival (PFS) (*p* < 0.05), suggesting that patients with high SLC1A5 expression had poorer OS and PFS than those with low expression (Fig. 1B, C). Furthermore, the expression of SLC1A5 was significantly higher in tumor stage III/IV compared to stage I/II patients, indicating a potential association with more malignant HCC (Fig. 1D).

### Alterations of SLC1A5 are correlated with TACE efficacy

**Fig. 2:**
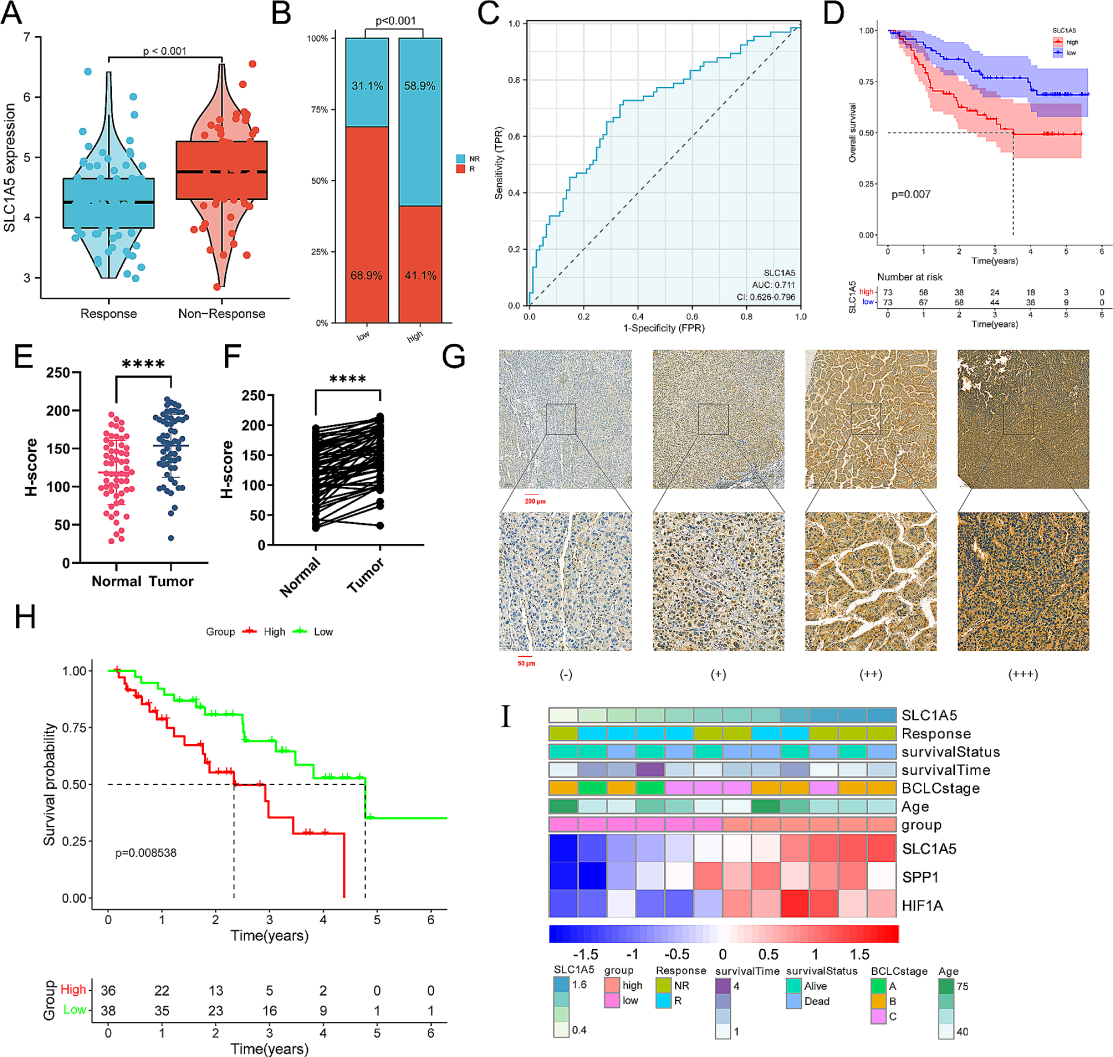
SLC1A5 correlated with TACE efficacy. (**A**) The difference expression of SLC1A5 between TACE non-response and response group. (**B**) The proportion of TACE response in different SLC1A5 expression groups (NR: no response; R: response). (**C**) The ROC curve for the predictive performance of SLC1A5 for TACE responsiveness. (**D**) K-M curves of TACE treated patients in different groups. (**E**) Overall comparison between HCC and adjacent tissues. (**F**) Paired comparison between HCC and adjacent tissues. (**G**) Representative immunohistochemically stained images of HCC tissues showing SLC1A5 expression (upper, 5.0×; lower, 20.0×). (**H**) The K-M curves of TACE treated patients in our externally validated cohort. (**I**) The heatmap of clinicopathological features and gene expressions

The GSE104580 dataset (comprising 81 responders and 66 non-responders HCC patients and related transcriptomic data) was procured to compare gene expression differences between TACE response and non-responders. In the GSE104580 dataset, SLC1A5 expression was higher in TACE non-responders than in responders (Fig. 2A). Interestingly, the non-responder proportion in the high expression group was significantly higher than in the low expression group (58.9% vs. 31.1%, *p* < 0.001) (Fig. 2B). Evaluation of SLC1A5’s predictive value on TACE responsiveness using diagnostic ROC curve demonstrated an AUC of 0.711 (95% CI: 0.626 ∼ 0.796) (Fig. 2C). 146 patients treated with TACE from the ICGC and GSE14520 datasets were classified into high- and low-expression groups based on the median SLC1A5 expression level. The detailed baseline characteristics of these 146 patients are summarized in Table 1. Survival analysis demonstrated that the survival time of the SLC1A5 low-expression group was notably longer than that of the high-expression group (*p* < 0.05) (Fig. 2D). The validation of 74 hepatocellular carcinoma patients who received TACE treatment in our center (detailed clinical information was displayed in Table 2) revealed that the staining intensity of SLC1A5 in HCC tissues was significantly higher than in adjacent normal tissues, with the staining intensity being inconsistent among HCC tissues (Fig. 2E-G). Likewise, survival analysis indicated that the overall survival (OS) of the low-expression group was considerably longer than that of the high group (*p* < 0.05) (Fig. 2H). A list of 12 patients with transcriptome sequencing data were divided into high- and low-expression groups based on the median SLC1A5 expression. The heatmap showed that patients with a higher SLC1A5 expression exhibited elevated expression of SPP1 and HIF1A, and were more likely to experience early death and TACE resistance compared to patients with low expression in our cohort (Fig. 2I).

**Table 1:** Baseline characteristics of TACE treated patients in different subgroups of the ICGC and GSE14520 datasets

Variables	Group	TACE cohort (*n* = 146)			*P* value
		High expression (*n* = 73)	Low expression (*n* = 73)		
Median Survival time (days)		757		1457	
Survival status	Alive	41		53	0.0568
	Dead	32		20	
Gender	Female	12		9	0.6380
	Male	61		64	
Age	≤ 60	52		48	0.5933
	> 60	21		25	
TNM stage	I	23		31	0.2382
	II	27		27	
	III	23		15	
BCLC stage	0	3		5	0.2339
	A	30		35	
	B	4		5	
	C	11		3	
	NA	25		25	
AFP (ng/ml)	< 300	24		27	0.6827
	≥ 300	24		21	
	NA	25		25	
Hepatitis B/C status	yes	47		45	0.6170
	no	1		3	
	NA	25		25	
Main tumor size (cm)	≤ 5	24		33	0.0959
	> 5	24		15	
	NA	25		25	
Multinodular	yes	11		7	0.4335
	no	37		41	
	NA	25		25	

**Table 2:** Baseline characteristics of 74 HCC patients treated with TACE in our cohort

Variables	Group	Our cohort (*n* = 74)		*P* value
		High expression (*n* = 36)	Low expression (*n* = 38)	
Median survival time (days)		475	704	
Survival status	Alive	23	23	0.8138
	Dead	13	15	
Gender	Female	3	3	> 0.9999
	Male	33	35	
Age	≤ 60	23	24	> 0.9999
	> 60	13	14	
AFP (ng/mL)	< 300	23	27	0.6211
	≥ 300	13	11	
Hepatitis B/C status	yes	32	36	0.4238
	no	4	2	
Main tumor size	≤ 5 cm	15	11	0.3312
	> 5 cm	21	27	
Multinodular	yes	17	14	0.4801
	no	19	24	
BCLC stage	A	12	9	0.5800
	B	15	20	
	C	9	9	

### SLC1A5 is positively correlated with hypoxia, angiogenesis, and immunosuppression

**Fig. 3:**
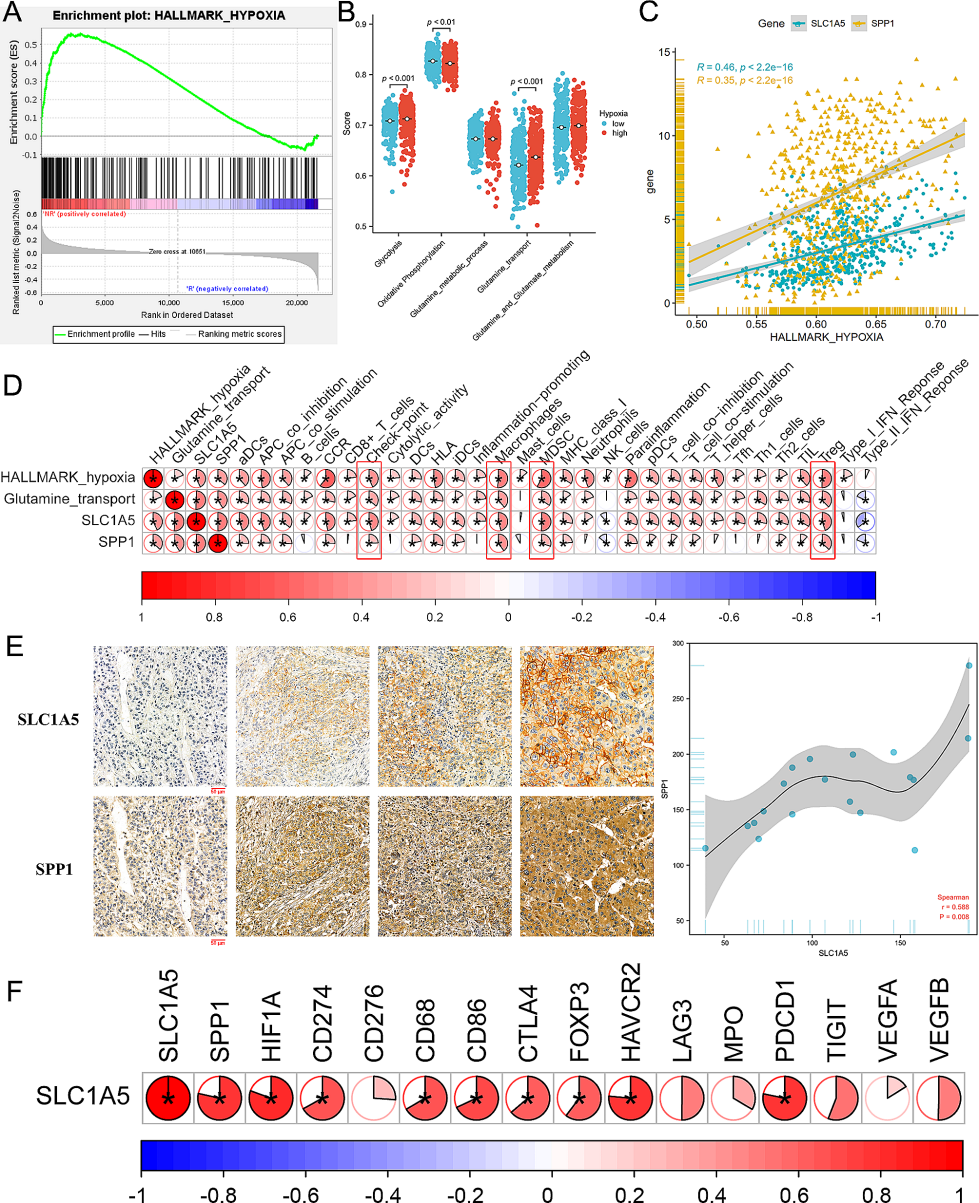
SLC1A5 correlated with hypoxia, angiogenesis, and immunosuppression. (**A**) TACE non-responsive group enriched in the hypoxia-related pathway. (**B, C**) Hypoxia could lead to an increase of glutamine transport and angiogenesis and SLC1A5 expression. (**D**) Heatmap showed the correlation among hypoxia, SLC1A5, SPP1, glutamine transport, and immune infiltration. (**E**) The left showed representative immunohistochemically stained images of SLC1A5 and SPP1 at the same tissue Sect. (20.0×). The right showed the correlation curve. (**F**) Heatmap showed SLC1A5 was associated with hypoxia, angiogenesis and immune status

Based on the finding in Fig. 3A, the non-responsive group of TACE could be enriched in hypoxic-related pathways. In light of these findings, it is speculated that the hypoxic conditions induced by TACE blocking blood flow could induce immune infiltration and metabolic alterations in tumors, thereby contributing to tumor recurrence and TACE resistance. Thus, we conducted a further analysis to investigate the correlation among hypoxia, glutamine transport and immune infiltration, as well as the relationship among SLC1A5 expression, hypoxia, and angiogenesis-related genes. Our ssGSEA results revealed that hypoxia could stimulate the upregulation of glutamine transport, angiogenesis, immunosuppressive cells infiltration and the expression of SLC1A5 (Fig. 3B-D). Furthermore, the expression of SLC1A5 was positively correlated with hypoxia and angiogenesis-related genes (Supplementary Fig. 1). Through the ssGSEA analysis, we observed a significant positive correlation between the expression of SLC1A5 and immune checkpoint pathways and immunosuppressive cells infiltration (macrophage, regulatory T cells (Tregs) and myeloid-derived suppressor cells (MDSCs)) (Fig. 3D). Notably, our immunohistochemistry, immunofluorescence, and transcriptome sequencing results of our cohort samples confirmed that the expression of SLC1A5 was positively linked with hypoxia, angiogenesis, immune checkpoint pathways, and immunosuppressive cell markers (Fig. 3E, F and Supplementary Fig. 1).

### SLC1A5 knockdown results in a less aggressive phenotype

**Fig. 4:**
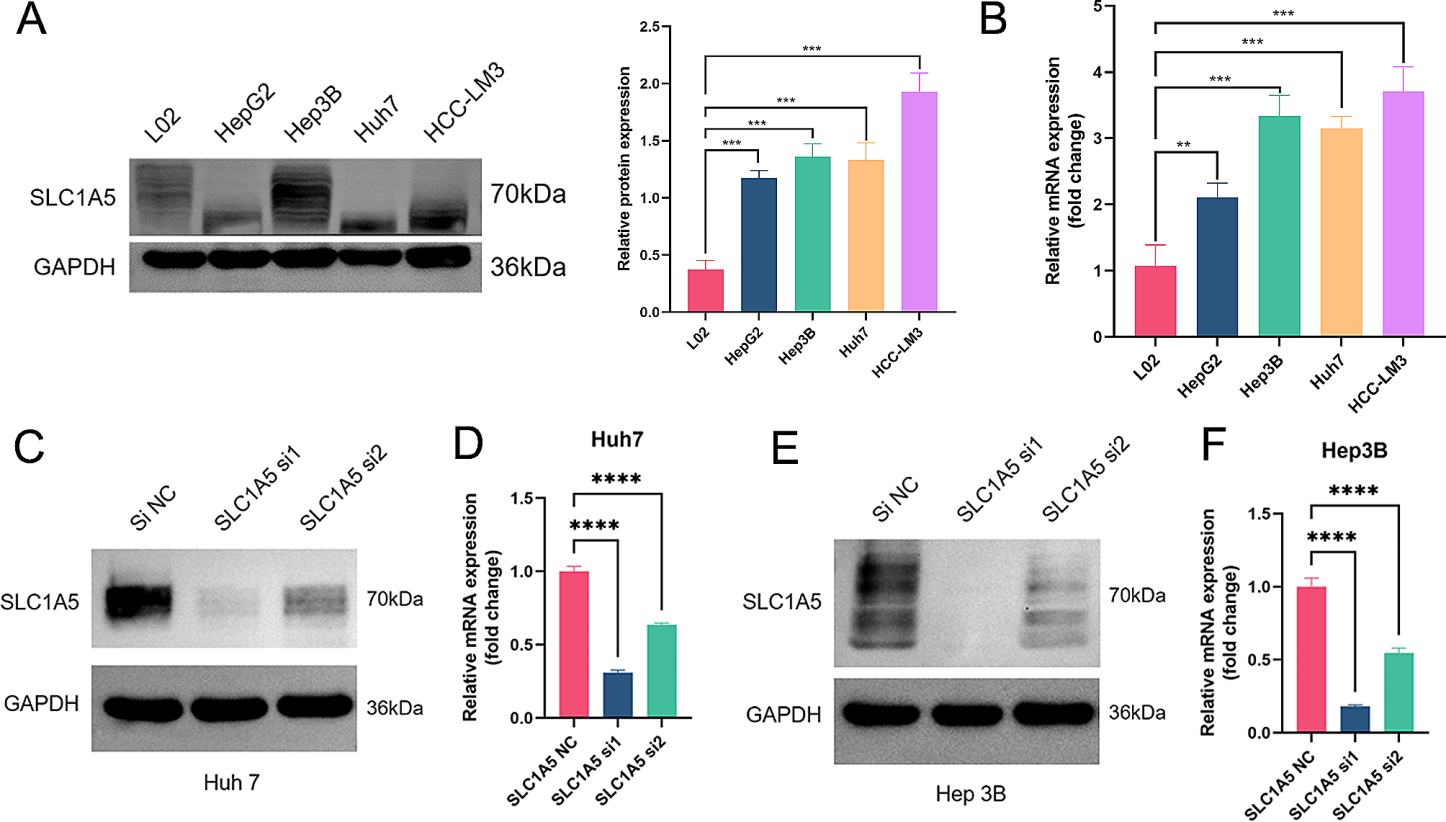
Construction of SLC1A5 knockdown liver cancer cell lines. (**A, B**) The WB and PCR detected SLC1A5 protein and mRNA. (**C, D**) The WB and PCR detected SLC1A5 protein and mRNA after knockdown in Huh7. (**E, F**) The WB and PCR detected SLC1A5 protein and mRNA after knockdown in Hep 3B

**Fig. 5:**
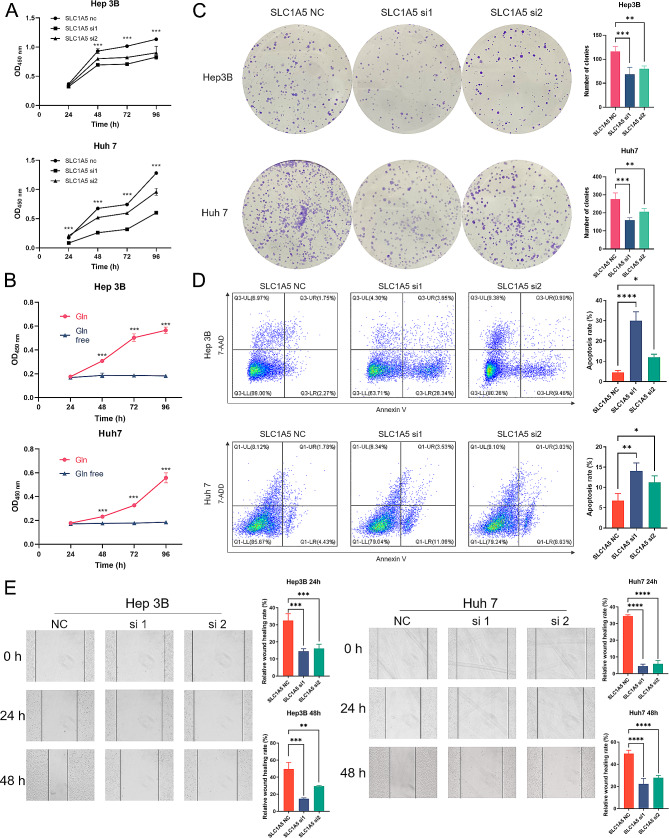
SLC1A5 Knockdown Affects Cancer Associated Phenotype. (**A**) The SLC1A5 effects on cell proliferation through the CCK8 assays. (**B**) Both Hep 3B and Huh7 cells ceased proliferation ability after deprivation of glutamine (**C**) The SLC1A5 effects on colony formation. (**D**) The SLC1A5 effects on cell apoptosis through the flow cytometry. (**E**) The SLC1A5 effects on cell migration through the wound-healing assays

Compared to the normal liver cell line L02, the SLC1A5 expression level was more substantially upregulated in liver cancer cells (HepG2, Hep 3B, Huh7, and HCC-LM3) (Fig. 4A, B). In this study, the Huh7 and Hep 3B cell lines with elevated SLC1A5 expression were selected for further experimentation. In order to allow and reduce the influence of chance errors and ensures the consistency of Knockdown results, Knockdown of SLC1A5 was constructed using sh-SLC1A5 lentivirus, which was divided into SLC1A5-NC, SLC1A5-si1, and SLC1A5-si2 groups. sh-SLC1A5 was able to effectively suppress SLC1A5 expression. The RT-qPCR and western blot results demonstrated that the SLC1A5 protein and mRNA expression levels in the SLC1A5-si1 and SLC1A5-si2 groups were markedly reduced compared to the SLC1A5-NC group, with SLC1A5-si1 exhibiting a more prominent decrease (Fig. 4C-F, and Supplementary Fig. 2A). In Huh7 and Hep 3B cells, SLC1A5 knockdown significantly hindered cell proliferation, colony formation, and migration while promoting apoptosis, with SLC1A5-si1 having a more pronounced impact (Fig. 5). Furthermore, it was observed that both Huh7 and Hep 3B cells ceased proliferating in glutamine-deprived medium (Fig. 5B).

### SLC1A5 knockdown suppressed tumor growth in Xenograft model and improved drug sensitivity

**Fig. 6:**
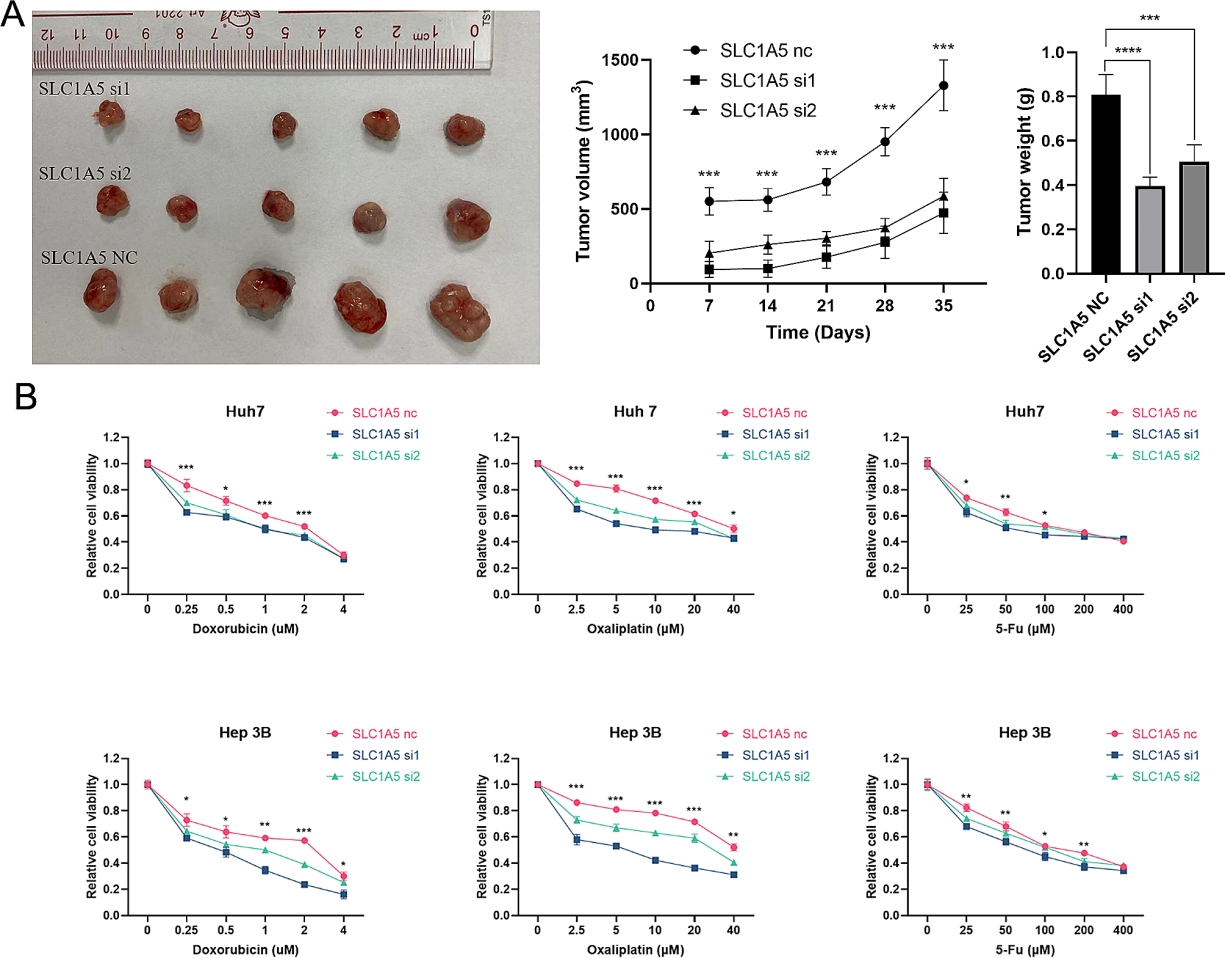
SLC1A5 Knockdown Suppressed Tumor Growth in Xenograft Model and Improved Drug Sensitivity. (**A**) SLC1A5 Knockdown Suppressed Tumor Growth in Xenograft Model. (**B**) SLC1A5 knockdown Sensitizes Cells to Chemotherapy

In *vivo* results demonstrated that SLC1A5 Knockdown significantly reduced the xenograft tumor growth. The tumor weight and volume in the sh-SLC1A5 group were significantly lower than those in the negative control group, with the SLC1A5-si1 group demonstrating the most significant decrease (Fig. 6A) (*p* < 0.05). We speculated that SLC1A5 knockdown may enhance the efficacy of TACE. We evaluated the impact of SLC1A5 knockdown on the cells’ response to doxorubicin, oxaliplatin, and fluorouracil, three commonly utilized chemotherapeutic drugs in TACE. Compared to the SLC1A5-NC group, the cell viability of the sh-SLC1A5 group was reduced under different concentrations of chemotherapeutic drugs (*p* < 0.05), with the SLC1A5-si1 group showing the most significant reduction. These findings suggested that SLC1A5 knockdown resulted in improved sensitivity of chemotherapy drugs in *vitro*.

### SLC1A5 activates EMT signaling pathway to promote cell migration and chemotherapeutic resistance

**Fig. 7:**
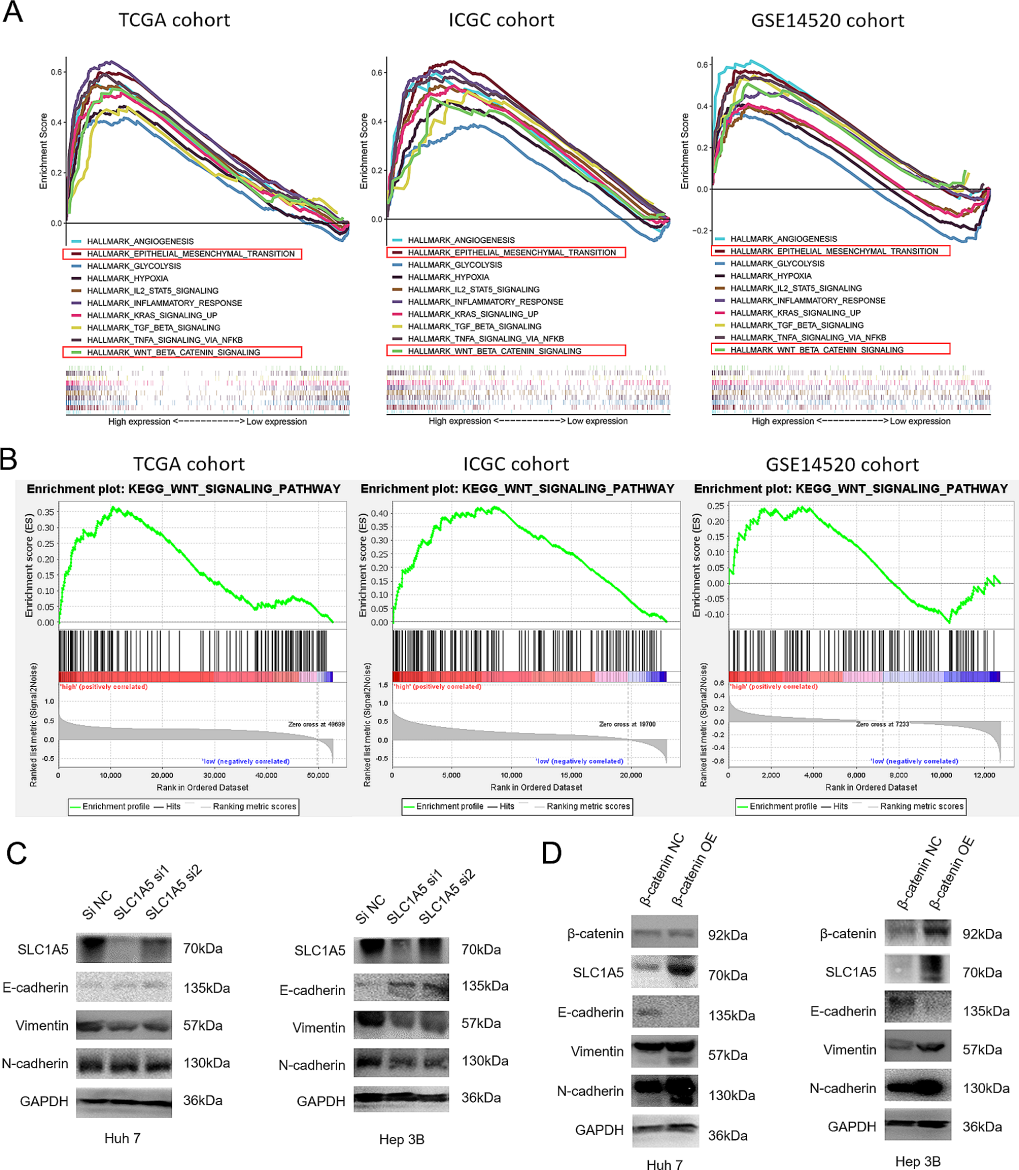
The effects of SLC1A5 on EMT signaling pathway. (**A, B**) GSEA analyzed the enrichment of SLC1A5 high-expression group in Hallmark and KEGG (*P* < 0.05, *FDR* < 0.25). (**C**) The protein expression of core molecules after SLC1A5 knockdown. (**D**) The protein expression of core molecules after overexpression of β-catenin in SLC1A5 knockdown Huh7 and Hep 3B cells

**Fig. 8:**
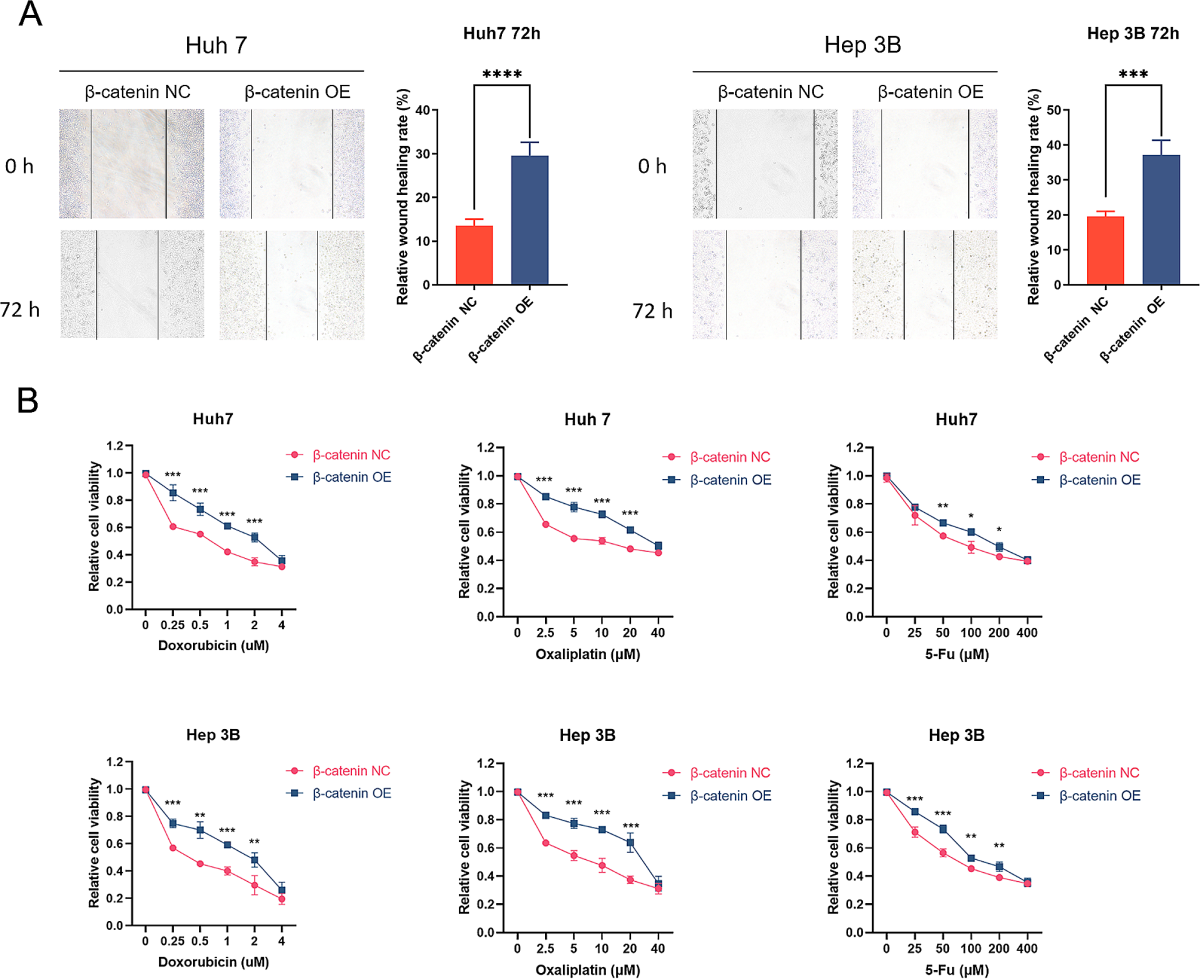
The effects of β-catenin overexpression on cell migration and chemotherapeutic resistance. (**A**) The inhibited cell migration phenotype of SLC1A5 knockdown was reversed by up-regulation of β-catenin expression. (**B**) The sensitizing cells to chemotherapy after SLC1A5 knockdown was reversed by up-regulation of β-catenin expression

We employed GSEA to predict the effects of SLC1A5 on biological signal pathways in HCC. Our findings revealed that hypoxia and angiogenesis signaling pathways are significantly enriched in high-SLC1A5-expression HCC samples. In addition, SLC1A5 overexpression positively correlated with multiple signaling pathways including Epithelial-mesenchymal transition (EMT), glycolysis, IL2-STAT5, inflammatory response, tumor growth factor, and Wnt/β-catenin signaling pathway (Fig. 7A). Furthermore, our KEGG enrichment results indicated that high expression of SLC1A5 was closely related to the Wnt signaling pathway (Fig. 7B). To assess the functional consequences of SLC1A5 downregulation, we conducted Western blot (WB) analysis on liver cancer cell lines. Results showed that following SLC1A5 knockdown in Huh7 and Hep 3B cells, Vimentin and N-cadherin expression were decreased, but E-cadherin expression was increased (Fig. 7C and Supplementary Fig. 3A, B). Moreover, overexpression of SLC1A5 in liver cancer cell line (PLC/PRF/5) significantly increased Vimentin and N-cadherin expression, while decreasing E-cadherin expression (Supplementary Fig. 2B). The relationship between SLC1A5 and EMT markers based on transcriptome sequencing data was showed in Supplementary Fig. 4, which indicated that SLC1A5 is highly correlated with Vimentin. Additionally, overexpression of β-catenin in SLC1A5 knockdown cell lines significantly increased SLC1A5, Vimentin and N-cadherin expression, while decreasing E-cadherin expression (Fig. 7D and Supplementary Fig. 3C, D). Moreover, our results indicated that overexpression of β-catenin in SLC1A5 knockdown cell lines significantly enhanced the migration ability and drug resistance in Huh7 and Hep 3B cells (Fig. 8).

## Discussion

In this study, a differential expression analysis was conducted on transcriptome data from three public hepatocellular carcinoma (HCC) datasets to investigate the expression of SLC1A5. As expected, the results revealed that the expression of SLC1A5 was significantly elevated in HCC, as confirmed by HCC samples obtained from our cohort. Additionally, the study determined that high expression levels of SLC1A5 were significantly associated with higher tumor (TNM) stage, suggesting its potential role in cancer progression and poor prognosis. Furthermore, survival analysis of patients with high SLC1A5 expression in HCC demonstrated that these individuals experienced significantly shorter overall survival and progression-free survival than those with low SLC1A5 expression, confirming that SLC1A5 expression levels could serve as an important prognostic indicator. Additionally, analysis of SLC1A5 expression and TACE treatment indicated that the expression of SLC1A5 in the TACE non-responder group was significantly higher than that in the TACE responder group, with a higher proportion of TACE non-responders in the SLC1A5 high expression group compared to the low expression group. These findings suggested that SLC1A5 expression may negatively influence TACE efficacy. Moreover, survival analysis from both the GSE14520 and ICGC datasets and our cohort demonstrated that patients with high SLC1A5 expression experienced significantly shorter survival after TACE treatment than those with low expression, indicating that SLC1A5 expression was correlated with the prognosis of TACE-treated patients. While SLC1A5 appears to be a promising candidate for use as a prognostic marker for HCC and TACE therapy, the specific mechanisms by which it influences HCC prognosis and TACE efficacy remain to be elucidated.

Our results indicated that hypoxia signaling was activated in patients with HCC who did not respond to TACE, supporting the assertion that TACE can induce tumor hypoxia. Previous research has demonstrated that hypoxia can stimulate altered tumor metabolism, including increased glutamine uptake [19, 27]. Hypoxia-inducible factor 1α (HIF-1α) is a key regulator of the hypoxic microenvironment and can influence tumor progression by regulating downstream transcription factors and signaling pathways [28]. Soluble protein phosphatase 1 (SPP1) is an angiogenic factor that is highly expressed in the tumor microenvironment under hypoxic conditions. Elevated SPP1 expression in the serum of HCC patients post-TACE has been linked to HCC recurrence and metastasis [29, 30]. Vascular endothelial growth factor (VEGF) is the principal regulator of tumor angiogenesis, and its levels are closely associated with cancer recurrence, metastasis, and patient prognosis. In this study, we observed that SLC1A5 expression was significantly correlated with HIF-1α, SPP1, and VEGF family members (VEGFA and VEGFB), consistent with the GSEA analysis demonstrating that high SLC1A5 expression is enriched in hypoxia and angiogenesis pathways. These findings suggested a strong association between SLC1A5 expression and hypoxia and angiogenesis. TACE-induced hypoxia has also been found to foster an immunosuppressive microenvironment. Our results indicated that SLC1A5 expression was significantly and positively correlated with immune checkpoint pathways, macrophage infiltration, regulatory T cells (Tregs), and other immunosuppressive factors. These findings supported a role for SLC1A5 in HCC prognosis and TACE resistance. TACE-induced hypoxia was associated with increased SLC1A5 expression, which promoted angiogenesis and immunosuppression, leading to recurrence and metastasis of residual cancer cells and limiting the effectiveness of TACE.

The expression of SLC1A5 was found to exert an influence on the malignant biological behavior of cancer cells through proliferation, cloning, migration, apoptosis, and xenograft tumor assays, reaffirming that the expression of SLC1A5 was closely related to progression and prognosis of HCC. Since SLC1A5 was correlated with TACE response, it was speculated that SLC1A5 expression was related to tumor cell sensitivity to chemotherapeutic agents, and that inhibition of SLC1A5 could improve TACE efficacy. Due to the use of chemotherapeutic agents such as doxorubicin, platinum, and fluorouracil in TACE, we have demonstrated that inhibition of SLC1A5 resulted in improved doxorubicin, oxaliplatin, and 5-fluorouracil sensitivity in HCC cell lines with an in *vitro* TACE assay. These results further supported the correlation between SLC1A5 and TACE response. However, the mechanism by which SLC1A5 may affect the sensitivity to chemotherapeutic agents is unclear.

Signaling pathway analysis by Gene Set Enrichment Analysis (GSEA) revealed a positive correlation between the high expression of SLC1A5 in HCC and the pathways of epithelial-mesenchymal transition (EMT) and Wnt/β-catenin. This suggested a potential involvement of SLC1A5 in malignant biological behavior of HCC cells through EMT and Wnt/β-catenin signaling pathways. EMT, a cellular process frequently associated with chemotherapy resistance in various types of tumors, is intricately regulated by a multitude of signaling pathways such as Wnt/β-catenin, transforming growth factor-β (TGF-β), Notch, and tyrosine kinase receptor (TKR) [31–33]. β-catenin, a critical component of Wnt/β-catenin signaling, has been shown to prevent apoptosis and enhance cell migration in HCC by triggering EMT, thereby contributing to drug and immune resistance [34]. Consistent with the literature and GSEA results, the present study proposed that SLC1A5 may mediate EMT in HCC cells, leading to enhanced invasive migration and drug resistance. To test this hypothesis, WB experiments were conducted, revealing that knockdown of SLC1A5 inhibited EMT signaling pathways, while overexpression was the opposite. Interestingly, overexpression of β-catenin in SLC1A5 knockdown cell lines reversed the effect of SLC1A5 knockdown on HCC cells, including upregulating SLC1A5 expression, confirming that Wnt/β-catenin may regulate SLC1A5 to induce EMT in HCC cells. Overall, SLC1A5 could promote the migration and drug resistance of liver cancer cells by upregulating EMT signaling pathway.

Although this study provides compelling evidence on the relationship between SLC1A5 and TACE treatment, further mechanistic studies could enhance the understanding of the specific pathways and mechanisms by which SLC1A5 affects the efficacy of TACE. The therapeutic principle of TACE is to block blood flow and chemotherapy. Therefore, the expression changes of SLC1A5 after TACE, especially its relationship with changes of microenvironment after TACE, require more in-depth research. In addition, the mechanism by which SLC1A5 leads to chemotherapy resistance needs to be confirmed in more comprehensive animal and clinical studies. These would be of particular interest to investigate in future studies.

## Conclusion

In conclusion, this study established that SLC1A5 was highly expressed in HCC and associated with tumor progression, poor prognosis, and resistance to TACE treatment. It was found that the expression of SLC1A5 was positively correlated with hypoxia, angiogenesis, and immunosuppressive infiltration, and hypoxia may induce upregulation of glutamine transport and SLC1A5 expression to promote angiogenesis and immunosuppressive infiltration. These elements may collaborate and interact with each other, thus affecting the efficacy and overall prognosis of TACE therapy. In addition, SLC1A5 could contribute to the migration and drug resistance of HCC cells by regulating EMT signaling pathway. Therefore, the modulation of glutamine transporter SLC1A5 could improve the efficacy of TACE therapy, potentially providing a new insight into combining TACE with therapies targeting tumor metabolism in HCC patients.

### Electronic supplementary material

Below is the link to the electronic supplementary material.

Supplementary Material 1

## Data Availability

Part of datasets involved in this study can be viewed in The Cancer Genome Atlas (TCGA, https://portal.gdc.cancer.gov/), International Cancer Genome Consortium (ICGC, https://dcc.icgc.org/projects/LIRI-JP), the Gene Expression Omnibus (GEO, https://www.ncbi.nlm.nih.gov/geo/query/acc.cgi?acc=GSE14520), and other datasets used and/or analyzed during the current study are available from the corresponding author on reasonable request.

## Abbreviations

AUC: Areas under the curve
DEGs: Differentially expressed genes
FDR: False discovery rate
GEO: Gene-Expression Omnibus
GSEA: Gene Set Enrichment Analysis
HCC: hepatocellular carcinoma
ICGC: International Cancer Genome Consortium
MDSCs: Myeloid-derived suppressor cells
OS: overall survival
PFS: Progression free survival
ROC: Receiver operator characteristic
SLC1A5: Solute Carrier Family 1 Member 5
ssGSEA: Single-sample gene-set enrichment analysis
TCGA: The Cancer Genome Atlas
