# Supplementary material for: Elevated SLC1A5 associated with poor prognosis and therapeutic resistance to transarterial chemoembolization in hepatocellular carcinoma
Source: J Transl Med. 2024 Jun 6;22:543. doi: 10.1186/s12967-024-05298-1 (PMC11157896; doi:10.1186/s12967-024-05298-1)
Supplement: Supplementary file 1 — Supplementary Material 1 [file 12967_2024_5298_MOESM1_ESM.docx]

**Elevated SLC1A5 Associated with Poor Prognosis and Therapeutic Resistance to Transarterial Chemoembolization in Hepatocellular Carcinoma**

**Supplementary Figures**


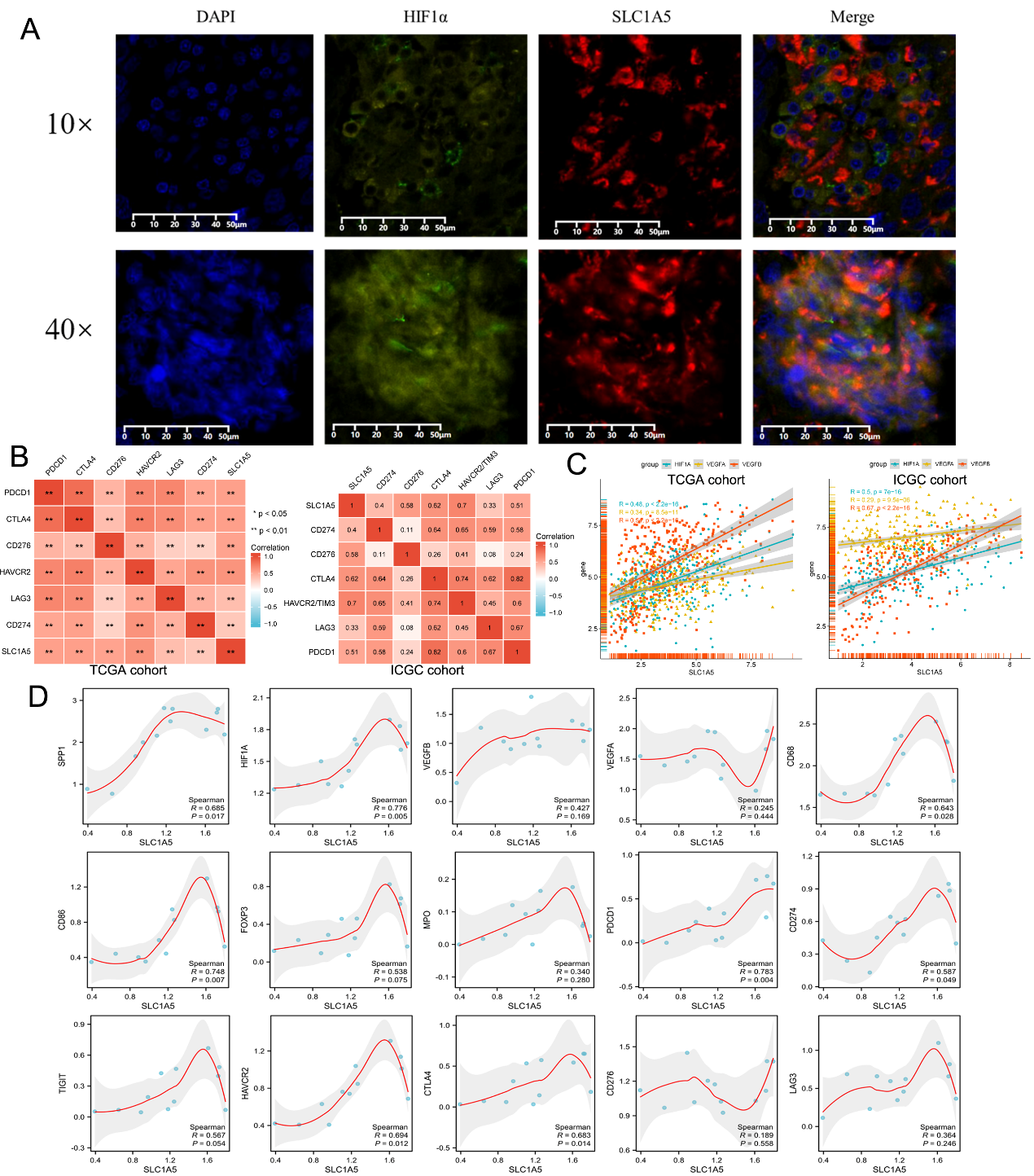


**Figure S1.** **SLC1A5 associated with hypoxia, angiogenesis, and immunosuppression.** (A) Representative immunofluorescence images of HCC tissues showing SLC1A5 and HIF-1α expression. (B) Heatmap showing the correlation between SLC1A5 and immune-checkpoint genes. (C) SLC1A5 closely related to the expression of HIF-1α, VEGFA and VEGFB. (D) Spearman’s rank correlation analysis of transcriptome sequencing in our cohort confirmed that the expression of SLC1A5 was associated with hypoxia, angiogenesis, immune checkpoint and immunosuppressive cell markers.


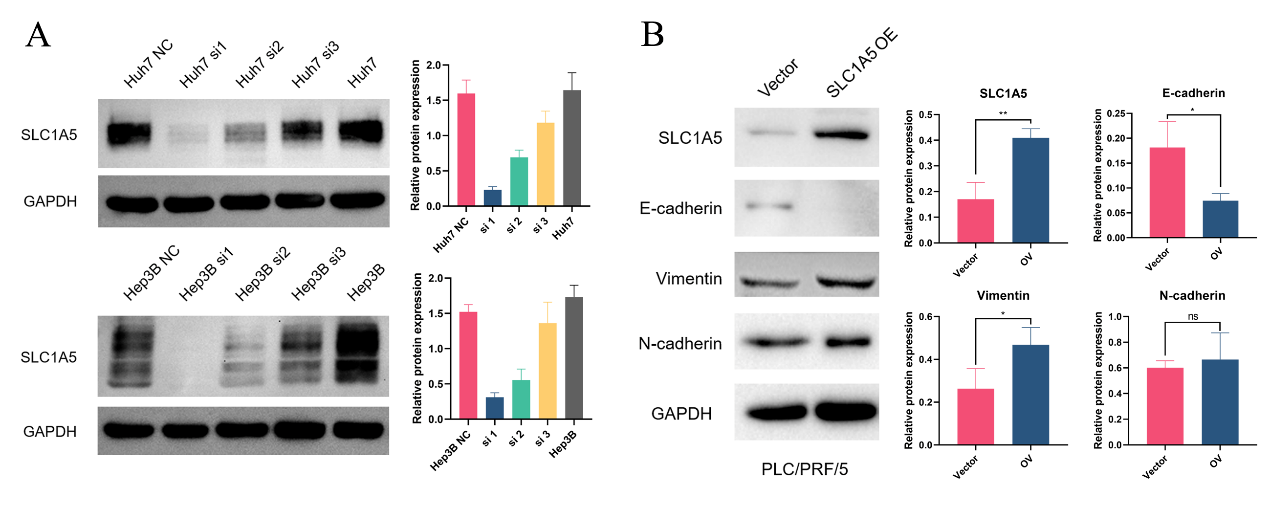


**Figure S2. Western blot test.** (A) The WB detected SLC1A5 protein in liver cancer cell lines (Complete image of Figure 4C and E). (B) The WB detected SLC1A5 protein after overexpression in PLC/PRF/5.


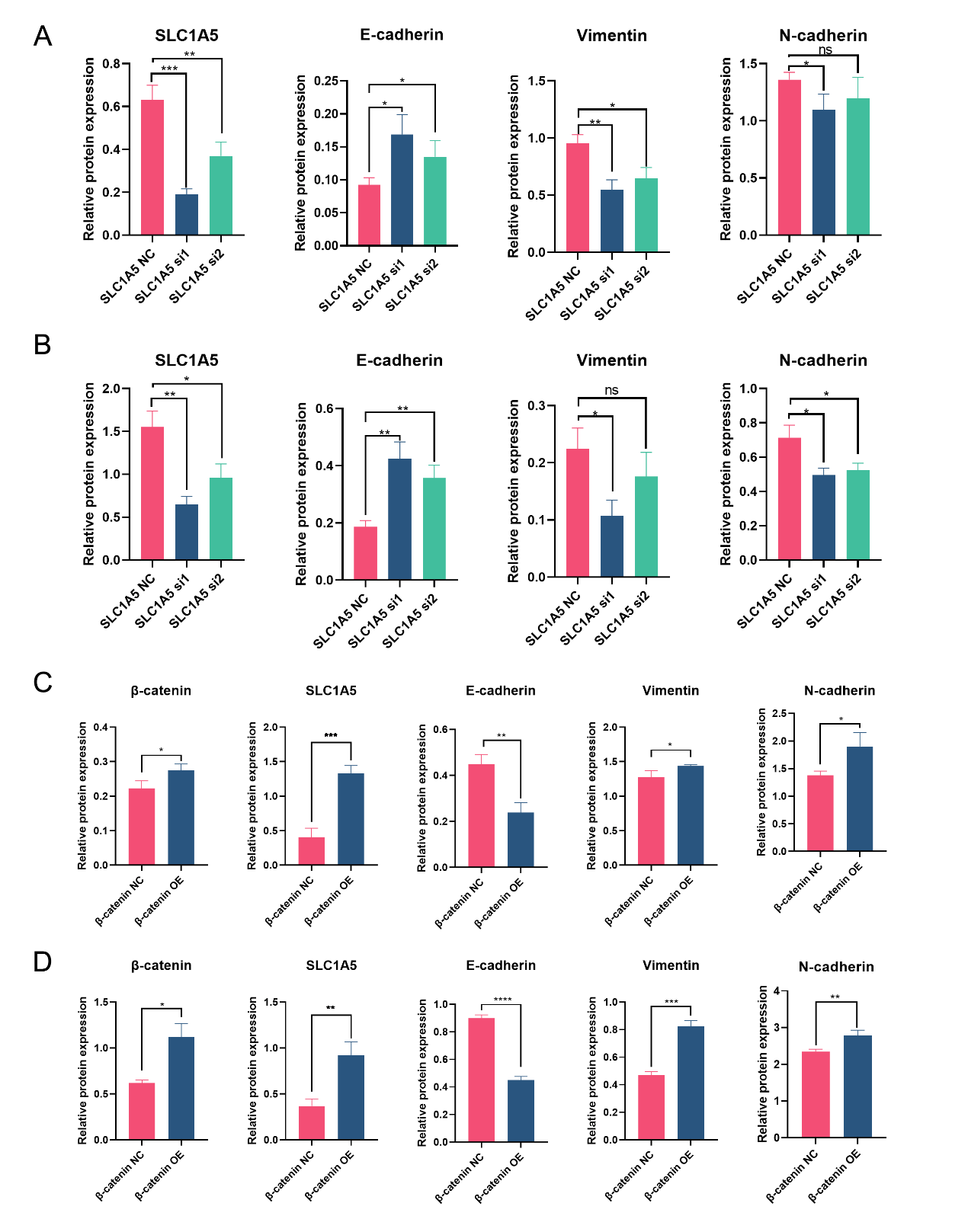


**Figure S3. The quantification of the signal obtained from the protein band in Figure 7C and D.** The quantification of the signal obtained from the protein band in Huh 7 and (B) Hep 3B cells after SLC1A5 knockdown. (D) The quantification of the signal obtained from the protein band after overexpression of β-catenin in SLC1A5 knockdown (C) Huh7 and (D) Hep 3B cells.


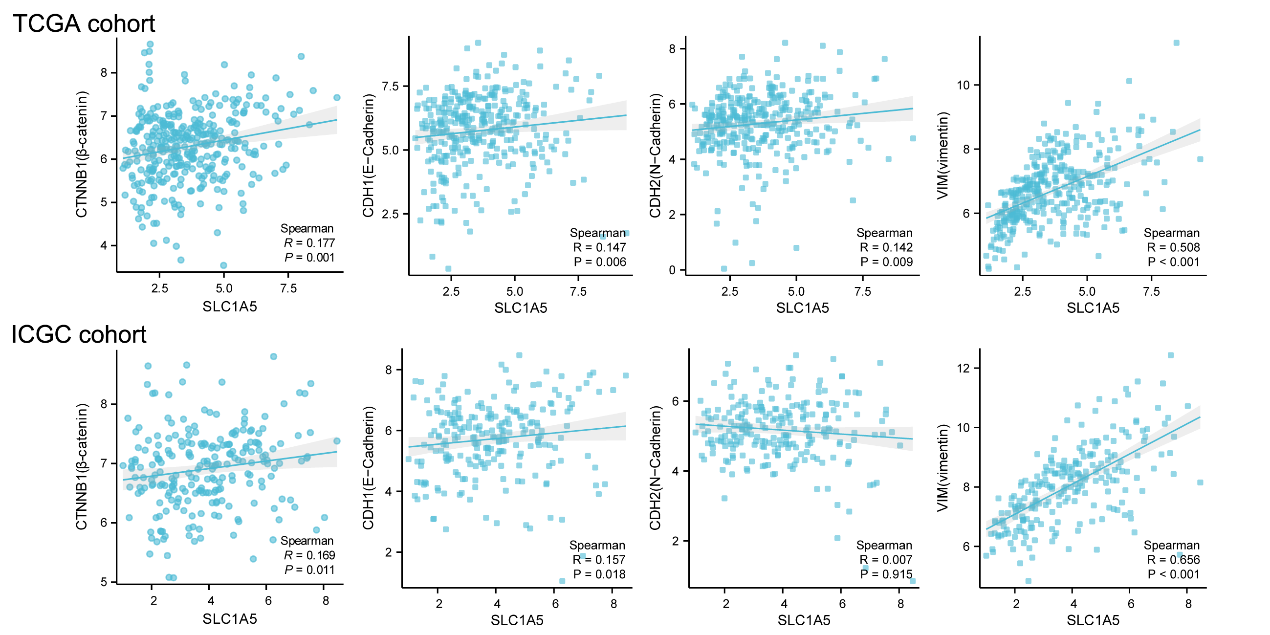


**Figure S4. The relationship between SLC1A5 and EMT markers based on transcriptome sequencing data.**

Table S1. Patient demographics and clinical characteristics of the included datasets.

| Variables | Group | TCGA cohort (n = 343) | ICGC cohort (n = 229) | GSE14520 cohort (n = 220) |
| --- | --- | --- | --- | --- |
|  |  |  |  |  |
| median Survival time (days) |  | 587 | 780 | 1570 |
|  |  |  |  |  |
| Survival status | Alive | 224 (65%) | 189 (83%) | 136 (62%) |
|  | Dead | 119 (35%) | 40 (17%) | 84 (38%) |
| Gender | Female | 110 (32%) | 61 (27%) | 30 (14%) |
|  | Male | 233 (68%) | 168 (73%) | 190 (86%) |
| Age | ≤60 | 165 (48%) | 49 (21%) | 181 (82%) |
|  | >60 | 178 (52%) | 180 (79%) | 39 (18%) |
| Tumor grade | G1 | 53 (15%) | / |  |
|  | G2 | 161 (47%) | / |  |
|  | G3 | 112 (33%) | / |  |
|  | G4 | 12 (3.5%) | / |  |
|  | Unknown | 5 (1.5%) | / |  |
| TNM staging | I | 161 (47%) | 36 (16%) | 93 (42%) |
|  | II | 77 (23%) | 105 (46%) | 77 (35%) |
|  | III | 80 (23%) | 69 (30%) | 48 (22%) |
|  | IV | 3 (1%) | 19 (8%) | / |
|  | Unknown | 22 (6%) | / | 2 (1%) |
